# A Multifunctional Polysaccharide Utilization Gene Cluster in *Colwellia echini* Encodes Enzymes for the Complete Degradation of κ-Carrageenan, ι-Carrageenan, and Hybrid β/κ-Carrageenan

**DOI:** 10.1128/mSphere.00792-19

**Published:** 2020-01-08

**Authors:** Line Christiansen, Duleepa Pathiraja, Pernille Kjersgaard Bech, Mikkel Schultz-Johansen, Rosanna Hennessy, David Teze, In-Geol Choi, Peter Stougaard

**Affiliations:** a Department of Plant and Environmental Sciences, University of Copenhagen, Copenhagen, Denmark; b Department of Biotechnology, College of Life Sciences and Biotechnology, Korea University, Seoul, South Korea; c DTU Bioengineering, The Technical University of Denmark, Lyngby, Denmark; University of British Columbia

**Keywords:** furcellaran, carrageenan, marine bacteria, algal polysaccharides, glycoside hydrolases, metabolic pathway

## Abstract

Algal cell wall polysaccharides constitute a large fraction in the biomass of marine primary producers and are thus important in nutrient transfer between trophic levels in the marine ecosystem. In order for this transfer to take place, polysaccharides must be degraded into smaller mono- and disaccharide units, which are subsequently metabolized, and key components in this degradation are bacterial enzymes. The marine bacterium Colwellia echini A3^T^ is a potent enzyme producer since it completely hydrolyzes agar and κ-carrageenan. Here, we report that the genome of C. echini A3^T^ harbors two large gene clusters for the degradation of carrageenan and agar, respectively. Phylogenetical and functional studies combined with transcriptomics and *in silico* structural modeling revealed that the carrageenolytic cluster encodes furcellaranases, a new class of glycoside hydrolase family 16 (GH16) enzymes that are key enzymes for hydrolysis of furcellaran, a hybrid carrageenan containing both β- and κ-carrageenan motifs. We show that furcellaranases degrade furcellaran into neocarratetraose-43-*O*-monosulfate [DA-(α1,3)-G4S-(β1,4)-DA-(α1,3)-G], and we propose a molecular model of furcellaranases and compare the active site architectures of furcellaranases, κ-carrageenases, β-agarases, and β-porphyranases. Furthermore, C. echini A3^T^ was shown to encode κ-carrageenases, *ι-*carrageenases, and members of a new class of enzymes, active only on hybrid β/κ-carrageenan tetrasaccharides. On the basis of our genomic, transcriptomic, and functional analyses of the carrageenolytic enzyme repertoire, we propose a new model for how C. echini A3^T^ degrades complex sulfated marine polysaccharides such as furcellaran, κ-carrageenan, and *ι-*carrageenan.

**IMPORTANCE** Here, we report that a recently described bacterium, Colwellia echini, harbors a large number of enzymes enabling the bacterium to grow on κ-carrageenan and agar. The genes are organized in two clusters that encode enzymes for the total degradation of κ-carrageenan and agar, respectively. As the first, we report on the structure/function relationship of a new class of enzymes that hydrolyze furcellaran, a partially sulfated β/κ-carrageenan. Using an *in silico* model, we hypothesize a molecular structure of furcellaranases and compare structural features and active site architectures of furcellaranases with those of other GH16 polysaccharide hydrolases, such as κ-carrageenases, β-agarases, and β-porphyranases. Furthermore, we describe a new class of enzymes distantly related to GH42 and GH160 β-galactosidases and show that this new class of enzymes is active only on hybrid β/κ-carrageenan oligosaccharides. Finally, we propose a new model for how the carrageenolytic enzyme repertoire enables C. echini to metabolize β/κ-, κ-, and *ι-*carrageenan.

## Abstract (precis)

Here, we report that a recently described bacterium, Colwellia echini, harbors a large number of enzymes enabling the bacterium to grow on κ-carrageenan and agar. The genes are organized in two clusters that encode enzymes for the total degradation of κ-carrageenan and agar, respectively. As the first, we report on the structure/function relationship of a new class of enzymes that hydrolyze furcellaran, a partially sulfated β/κ-carrageenan. Using an *in silico* model, we hypothesize a molecular structure of furcellaranases and compare structural features and active site architectures of furcellaranases with those of other GH16 polysaccharide hydrolases, such as κ-carrageenases, β-agarases, and β-porphyranases. Furthermore, we describe a new class of enzymes distantly related to GH42 and GH160 β-galactosidases and show that this new class of enzymes is active only on hybrid β/κ-carrageenan oligosaccharides. Finally, we propose a new model for how the carrageenolytic enzyme repertoire enables C. echini to metabolize β/κ-, κ-, and *ι-*carrageenan.

## INTRODUCTION

Primary production in the marine environment adds up to approximately half of the primary production on Earth (1). Thus, degradation of marine phytoplankton and of macrophytes (seaweed and sea grasses) is important in order for nutrients to be transferred between trophic levels in the marine food web; in particular, cell wall polysaccharides constitute a large fraction of the biomass of marine primary producers (2, 3). The compositions of cell walls from marine algae share several features with those of land plants: Both plant groups have cellulose and hemicellulose in their cell walls, but in contrast to land plants, marine algae contain a variety of sulfated polysaccharides, e.g., agar, porphyran, and carrageenan in red algae; ulvan in green algae; and fucan in brown algae (4). Consequently, in order for organisms to degrade algal polysaccharides, they must produce enzymes that are active on the respective sulfated polysaccharides (5).

Like terrestrial herbivores, marine herbivores harbor endosymbiotic microorganisms that produce enzymes needed for the hydrolysis of plant cell wall polysaccharides, notably, cellulose and hemicellulose but also more-complex sulfated carbohydrates. In marine iguanas, the fecal microbiota produce enzymes specific for utilization of marine polysaccharides (6), turban shell (Batillus cornutus) feeding on brown algae harbor intestinal bacteria producing cellulases, alginate lyases, laminarinases, and “kelp lyases” (7), and alginolytic bacteria may be found in the gut of sea urchins (*Strongylocentrotus* sp.) and abalones (*Haliotis* sp.) (8).

We have previously isolated a bacterium, Colwellia echini A3^T^, from the intestine of the sea urchin Strongylocentrotus droebachiensis and found that this bacterium solubilizes agar and carrageenan plate media as a result of enzymatic hydrolysis (9). Here, we demonstrate that C. echini harbors two large multifunctional polysaccharide utilization loci (PULs) encoding genes for the total catabolism of not only agar and κ-carrageenan but also *ι-*carrageenan and the hybrid β/κ-carrageenan furcellaran. Furthermore, we show that furcellaran degradation by C. echini is catalyzed by enzymes belonging to a new GH16_13 subfamily that are similar to those previously reported for another marine bacterium, Paraglaciecola hydrolytica S66^T^ (10, 11). Using transcriptomics, *in silico* analyses, and recombinant enzyme technology, we provide more information about the reaction mechanism of GH16_13 furcellarases and characterize the oligosaccharide products that they release. Finally, we present a model for how C. echini A3^T^ degrades furcellaran, κ-carrageenan, and *ι-*carrageenan. The results presented here not only improve our understanding of degradation of sulfated marine polysaccharides but also may promote the generation of sulfated oligosaccharides to be used in biotechnology and pharma.

## RESULTS

### Polysaccharide degradation potential of Colwellia echini A3^T^.

Bioinformatic analyses showed that the genome of C. echini encodes a large number of carbohydrate-active enzymes (CAZymes) (12). In comparisons to phylogenetically related *Colwellia* species, C. echini A3^T^ encoded the largest amount of CAZymes (see Table S1A in the supplemental material). In accordance with the agarolytic nature of C. echini A3^T^ and C. agarivorans QM50^T^ (13), these two bacterial species encoded enzymes belonging to GH families with predominantly agar-degrading representatives such as GH50 (β-agarases), GH86 (β-agarases/porphyranases), and GH117 (α-3,6-anhydrogalactosidases), which are keystone enzymes in and indicative of agar catabolism (14). In contrast, such genes were absent in genome sequences of the nonagarolytic relatives C. psychrerythraea 34H^T^, C. piezophila Y223G^T^, and C. chukchiensis BCw111^T^ (Table S1B). Unique for C. echini was the presence of GH96 α-agarases, which are rarely reported and include only a few characterized members (15–18). Of all the *Colwellia* genomes analyzed, that of C. echini encoded the largest amount of GH16 CAZymes, comprising β-agarases, β-porphyranases, laminarinases, and κ-carrageenases, and only C. echini encoded GH82 *ι-*carrageenases.

10.1128/mSphere.00792-19.5 TABLE S1 (A) Number of genes encoding CAZy family proteins in *Colwellia* species. AA, auxiliary activities; CBM, carbohydrate binding modules; CE, carbohydrate esterases; GH, glycosyl hydrolases; GT, glycosyl transferases; PL, polysaccharide lyases; Sulf, formylglycine-dependent sulfatases. (B) Number of red algal polysaccharide-specific CAZymes in *Colwellia* species. Download Table S1, DOCX file, 0.01 MB. Copyright © 2020 Christiansen et al. 2020 Christiansen et al. This content is distributed under the terms of the Creative Commons Attribution 4.0 International license.

**FIG 1:**
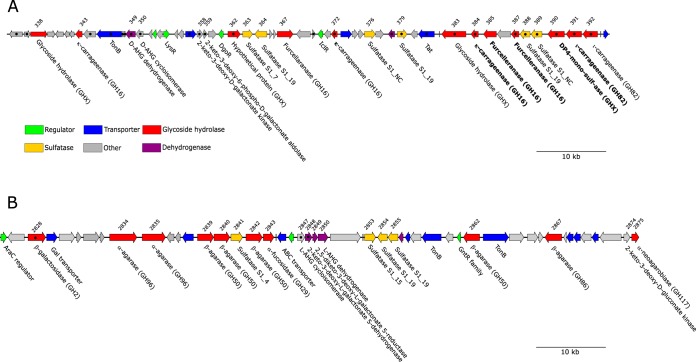
Map of the carrageenolytic CAR PUL (A) and of the agarolytic AGA PUL (B). Arrows indicate open reading frames (ORFs), and the color code shows the predicted or documented function of the ORFs. Gene functions documented in this work are shown in bold, and genes where the function was predicted by bioinformatic analyses are shown in regular font. ORF numbers are indicated above the gene map. Black dots inside ORFs indicate that the gene was upregulated more than 2 log in transcriptomics analysis when C. echini A3^T^ was cultivated with κ-carrageenan.

The genes that encode putative agarases and carrageenases were localized in two large gene clusters: one ∼92,000-bp region dedicated to agar degradation and one ∼86,000-bp region dedicated to carrageenan degradation (Fig. 1). TonB-dependent receptors, previously proposed to be analogous to the *Bacteroidetes* detection system (10, 19, 20), were located in both gene clusters (Ce2863 and Ce345; Fig. 1). Furthermore, as putative transporters were similarly located in the gene clusters, we hypothesize that the two regions could be analogous to the polysaccharide utilization loci (PULs) originally described in *Bacteriodetes* (19). Thus, we named the gene cluster encoding agarolytic enzymes AGA PUL and the cluster with carrageenolytic genes CAR PUL (Fig. 1).

### Glycoside hydrolases in the CAR PUL gene cluster.

**FIG 2:**
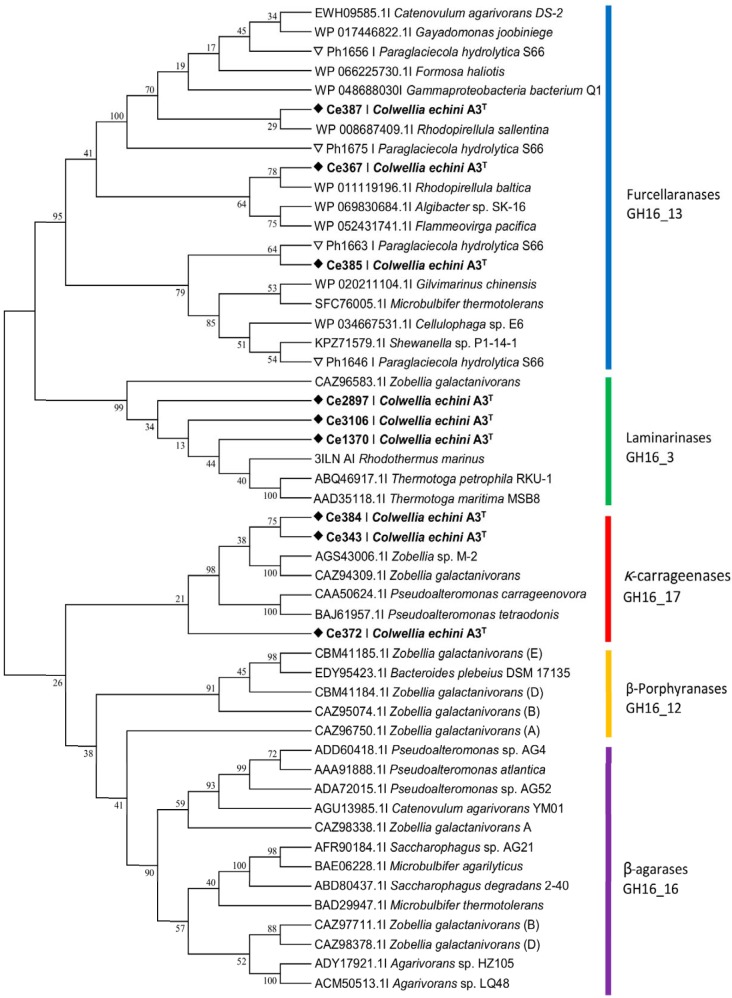
Phylogenetic tree of GH16 enzymes from C. echini A3^T^. Annotated β-agarases, κ-carrageenases, laminarinases, and β-porphyranases from C. echini A3^T^ (black diamonds) were analyzed together with characterized proteins from the CAZy database. Furcellaranases from C. echini A3^T^ were compared to characterized furcellaranases from P. hydrolytica (white triangles) and sequence homologs derived from the NCBI database. The tree was constructed by using the neighbor-joining method. Bootstrap values represent percentages of 1,000 replications.

An automatic annotation showed that CAR PUL encoded three putative GH16 β-porphyranases (Ce367, Ce385, and Ce387), three putative GH16 κ-carrageenases (Ce343, Ce372, and Ce384), and two putative GH82 *ι-*carrageenases (Ce391 and Ce392) (Fig. 1A; see also Table S2). However, the level of identity between the putative glycoside hydrolases encoded by CAR PUL and sequences in the Protein Data Bank database (PDB) or Swiss-Prot database was low (Table S2). Therefore, a phylogenetic analysis of the glycoside hydrolases was initiated. GH16 sequences with known function from the PDB database and sequences from recently characterized, novel furcellaranases from P. hydrolytica S66^T^ (10) were compared to GH16 sequences from CAR PUL (Fig. 2). Enzymes Ce367, Ce385, and Ce387 grouped with the newly characterized GH16_13 furcellaranases Ph1656, Ph1663, and Ph1675 from P. hydrolytica S66^T^ (10). Ce387 showed 57% identity to Ph1656 and 46% identity to Ph1675, whereas Ce367 and Ce385 displayed less than 20% identity to the furcellaranases from P. hydrolytica S66^T^. Also shown in Fig. 2 is the clustering of Ce343 and Ce384 with known κ-carrageenases. Ce372 clustered distantly with κ-carrageenases, but as the bootstrap value was very low (21%) and since we could not produce active enzyme, no activity could be assigned to Ce372. In addition, Ce1370, Ce2897, and Ce3106, located outside CAR PUL, clustered with known laminarinases. No enzyme sequences from strain A3^T^ grouped with sequences of characterized GH16_12 β-porphyranases or GH16_16 β-agarases (Fig. 2). A closer analysis of the AGA PUL region showed the presence of GH50 and GH86 β-agarases and the rare GH96 α-agarases, which could be the reason for the observed agarolytic activity; this activity is to be described in detail elsewhere.

10.1128/mSphere.00792-19.6 TABLE S2 Identity of CAZymes encoded by CAR PUL in C. echini A3. Amino acid sequences from each of the genes were analyzed by BLASTp using sequences in the NCBI Protein Data Bank (PDB) and nonredundant protein sequences. Download Table S2, DOCX file, 0.01 MB. Copyright © 2020 Christiansen et al. 2020 Christiansen et al. This content is distributed under the terms of the Creative Commons Attribution 4.0 International license.

**FIG 3:**
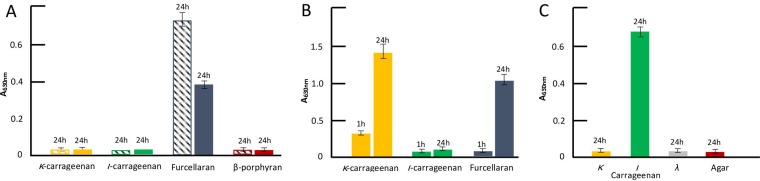
Substrate specificity of (A) furcellaranases (Ce385, dashed; Ce387, solid), (B) κ-carrageenase (Ce384), and (C) *ι-*carrageenase (Ce391). Agar, furcellaran, porphyran, κ-carrageenan, *ι-*carrageenan, and/or *λ*-carrageenan were used as substrates. The substrate concentration was 0.1% (wt/vol) in all enzyme reactions. The amount of reducing ends was determined by 3-methyl-2-benzothiazolinone hydrazone (MBTH) assay, and the number of aldehydes in the reducing end of carbohydrates was measured as absorbance at 630 nm (A_630nm_). All assays were carried out in triplicate at 20°C and terminated after 1 h (1h) and/or 24 h (24h).

**FIG 4:**
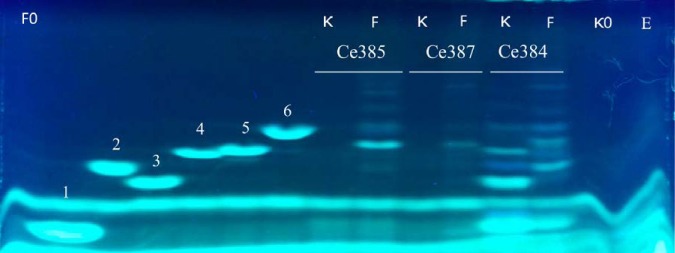
FACE analysis of enzymatic hydrolysis of 0.1% (wt/vol) κ-carrageenan (K) and 0.1% (wt/vol) furcellaran (F). Furcellaranases Ce385 and Ce387 and κ-carrageenase Ce384 were incubated with furcellaran or κ-carrageenan at 20°C overnight. Reference standards without enzyme as indicated as follows: 1, neocarrabiose-monosulfate (DP2-monoS); 2, neocarratetraose-monosulfate (DP4-monoS); 3, neocarratetraose-disulfate (DP4-diS); 4, neocarrahexaose-trisulfate (DP6-triS); 5, neocarrahexaose-tetrasulfate (DP6-tetraS); 6, neocarraoctaose-tetrasulfate (DP8-tetraS). F0, furcellaran without enzymes; κ0, κ-carrageenan without enzymes; E, mixture of all enzymes and no carbohydrate substrate.

Functional analyses were carried out in order to confirm the substrate specificity of Ce367, Ce385, and Ce387. Recombinant, His-tagged Ce385 and Ce387 were produced in Escherichia coli BL21, purified with nickel-nitrilotriacetic acid (Ni-NTA) chromatography, and assayed with furcellaran, porphyran, and κ-and *ι-*carrageenan as the substrates; Ce367 could not be expressed as a soluble and active enzyme in this study. Substrate specificities of the recombinant enzymes were analyzed in a reducing sugar assay (Fig. 3A) and by fluorophore-assisted carbohydrate electrophoresis (FACE) (Fig. 4). The results showed that Ce385 and Ce387 hydrolyzed furcellaran, a partially sulfated hybrid β/κ-carrageenan (21, 22), but not (more highly sulfated) κ-or *ι-*carrageenan or porphyran. No activity was detected with agar or agarose, and no activity on any substrates was detected in extracts from E. coli with empty vector (not shown). Matrix-assisted laser desorption ionization–time of flight (MALDI-TOF) analysis showed that the hydrolysis products seen with furcellaran as the substrate were neocarratetraose monosulfate and neocarrahexaose monosulfate (see Fig. S3 in the supplemental material).

### *In silico* specificity study of furcellaranases.

From the structure of substrates and products of furcellaranase-catalyzed reactions, a cleavage event leaving either a G4S or a galactose (Gal) at the reducing end could be envisioned. Remarkably, the GH16 family comprises enzymes that are able to catalyze reactions leading to products presenting a sulfate at the fourth hydroxyl of the reducing-end sugar (e.g., κ-carrageenases) or an unsulfated sugar (e.g., β-agarases and β-porphyranases), and the molecular determinants allowing these two distinct specificities have previously been highlighted. Indeed, in known κ-carrageenase structures (PDB 5OCR, Zobellia galactanivorans; PDB 5OCQ and 1DYP, P. carrageenovora), an arginine and a tryptophan are responsible for binding to the sulfate of G4S in the –1 subsite; while a glutamate was reported to be a critical residue for recognition of the fourth hydroxyl of nonsulfated d-galactosidase (d-Gal) in the –1 subsite of both β-porphyranases (3JUU and 3ILF, Z. galactanivorans; 4AWD, Bacteroides plebeius) and β-agarases (3WZ1, Marinactinospora thermotolerans; 1O4Y, Z. galactanivorans) (23–25). To assess whether furcellaranases presented one of these residue sets, 2,226 GH16 sequences, sharing 10% to 80% pairwise identity and including characterized representatives for β-agarase, κ-carrageenase, porphyranase, and furcellaranase activities, were aligned by iterative multiple-sequence alignment. The characteristic GH16 catalytic triad ExDxxE was found to be largely conserved (99.5%, 97.2%, and 99.2%, respectively, for each catalytic residue). The glutamate recognizing nonsulfated Gal, together with a tryptophan in an ExxxW motif, was found in 65% of the sequences, including all the β-agarase, porphyranase, and furcellaranase sequences (Table 1). As GH16 catalysis proceeds through important conformational changes of the sugar in the –1 subsite, binding a distorted ^1^S_3_ skew-boat conformation (26), it requires particularly strong interaction network at this site. Therefore, it is likely that furcellaranases accommodate a nonsulfated Gal in their –1 subsite. *In silico* folding of the C. echini furcellaranases and κ-carrageenase over known structures of GH16 showed that in all cases, the catalytic triad ExDxxE and the platform Trp that precedes it, as well as the pairs of residues pinpointed above as recognizing either Gal or G4S, superimposed well (Fig. S1).

**TABLE 1:** Key residues in the −1 subsites of algal cell wall-degrading GH16 enzymes

Enzyme-activity	Organism	PDB code(s) or name	Platform	Catalytic residues	−1 sugar	4 interacting residues
κ-Carrageenase	Z. galactanivorans	5OCR	Trp^141^	Glu^159^, Asp^161^, Glu^164^	G4S	Trp^74^, Arg^263^
	P. carrageenovora	5OCQ, 1DYP	Trp^144^	Glu^163^, Asp^165^, Glu^168^	G4S	Trp^67^, Arg^260^
	C. echini	Ce343 (model)	Trp^155^	Glu^180^, Asp^182^, Glu^185^	G4S	Trp^78^, Arg^286^
	C. echini	Ce384 (model)	Trp^189^	Glu^214^, Asp^216^, Glu^219^	G4S	Trp^110^, Arg^324^

β-Agarase	M. thermotolerans	3WZ1	Trp^138^	Glu^147^, Asp^149^, Glu^152^	Gal	Glu^257^
	Z. galactanivorans	1O4Y	Trp^138^	Glu^147^, Asp^149^, Glu^152^	Gal	Glu^254^

β-Porphyranase	Z. galactanivorans	3JUU (PorB)	Trp^139^	Glu^156^, Asp^158^, Glu^161^	Gal	Glu^256^
	Z. galactanivorans	3ILF (PorA)	Trp^131^	Glu^139^, Asp^141^, Glu^144^	Gal	Glu^234^
	B. plebeius	4AWD	Trp^149^	Glu^173^, Asp^175^, Glu^178^	Gal	Glu^284^

Furcellaranase	C. echini	Ce385 (model)	Trp^139^	Glu^146^, Asp^148^, Glu^151^	Gal	Glu^271^
	C. echini	Ce387 (model)	Trp^202^	Glu^209^, Asp^211^, Glu^214^	Gal	Glu^320^
	P. hydrolytica	Ph1631 (model)	Trp^137^	Glu^146^, Asp^148^, Glu^151^	Gal	Glu^254^
	P. hydrolytica	Ph1663 (model)	Trp^149^	Glu^156^, Asp^158^, Glu^161^	Gal	Glu^255^
	P. hydrolytica	Ph1675 (model)	Trp^176^	Glu^193^, Asp^195^, Glu^198^	Gal	Glu^305^

10.1128/mSphere.00792-19.1 FIG S1 Superimposition of C. echini enzyme models and known GH16 structures. (A) Furcellaranases Ce385 and Ce387 are shown in purple and blue, respectively, superimposed with porphyranases (3ILF and 3JUU, Z. galactanivorans, green and light green, respectively) and agarase (3WZ1, *M. thermotolerans*, dark green). Numbering is according to Z. galactanivorans PorB (3JUU). (B) κ-Carrageenase Ce343 (salmon) and Ce384 (green) models, superimposed with κ-carrageenase experimental structures from P. carrageenovora (1DYP, light blue) and Z. galactanivorans (5OCR, yellow). Numbering is according to Z. galactanivorans 5OCR. Download FIG S1, PDF file, 0.3 MB. Copyright © 2020 Christiansen et al. 2020 Christiansen et al. This content is distributed under the terms of the Creative Commons Attribution 4.0 International license.

### Functional characterization of carrageenan degradation.

Phylogenetic analysis indicated that Ce343 and Ce384 are representatives of GH16_17 κ-carrageenases (Fig. 2). Functional studies showed that recombinant Ce384 produced in E. coli hydrolyzed κ-carrageenan and the hybrid β/κ-carrageenan furcellaran but not *ι-*carrageenan (Fig. 3B; see also Fig. 4) or agar or agarose (not shown). MALDI-TOF analysis showed that Ce384 degrades κ-carrageenan into neocarrabiose monosulfate (DA-G4S). Oligosaccharide products released from furcellaran were neocarrabiose monosulfate (DA-G4S), neocarratetraose monosulfate (DA-G4S-DA-G or DA-G-DA-G4S), and neocarrahexaose monosulfate (DA-G4S-DA-G-DA-G or DA-G-DA-G4S-DA-G or DA-G-DA-G-DA-G4S) (Fig. S3). *In silico* modeling showed that κ-carrageenases Ce343 and Ce384 superimposed with κ-carrageenase experimental structures from P. carrageenovora (1DYP) and Z. galactanivorans (5OCR) (Fig. S1).

**FIG 5:**
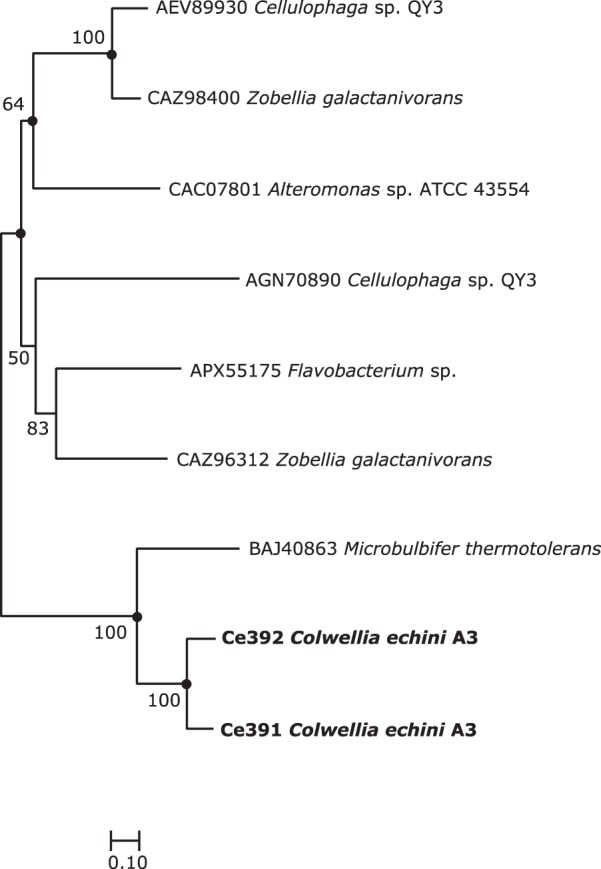
Neighbor-joining tree based on amino acid sequences of *ι-*carrageenases Ce391 and Ce392 from C. echini A3^T^ and related sequences retrieved from the NCBI database. Numbers at nodes are bootstrap values and represent percentages of 1,000 replicates; only values of >50% are shown. Filled circles indicate that the corresponding nodes were also recovered in the tree generated with the maximum likelihood algorithm. Bar, 0.1 changes per position.

**FIG 6:**
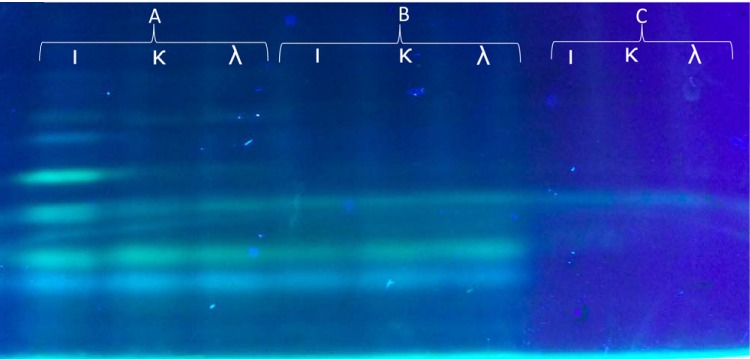
*ι-*Carrageenase activity visualized by FACE. 0.1% (wt/vol) *ι-*, λ-, or κ-carrageenan (ι, λ, or κ) incubated at 20°C overnight (A) with recombinant Ce391 enzyme extracts or (B) with extracts from E. coli with empty vector or (C) without enzyme extracts.

Ce391 and Ce392 displayed low-level phylogenetic relationships (14% to 19% identity) to known *ι-*carrageenases in the PDB and Swiss-Prot databases (Fig. 5; see also Table S2). Biochemical analyses showed that recombinant Ce391 degrades *ι-*carrageenan but not agar, furcellaran, or κ- or *λ-*carrageenan (Fig. 3C; see also Fig. 6). Negative controls with extracts from empty vector containing E. coli showed no degradation of any of the polysaccharides. The band pattern in the FACE gel indicated that the products were neocarrabiose disulfate (DA2S-G4S) and possibly neocarratetraose tetrasulfate (DA2S–G4S-DA2S–G4S).

### Miscellaneous CAZymes in CAR PUL.

**FIG 7:**
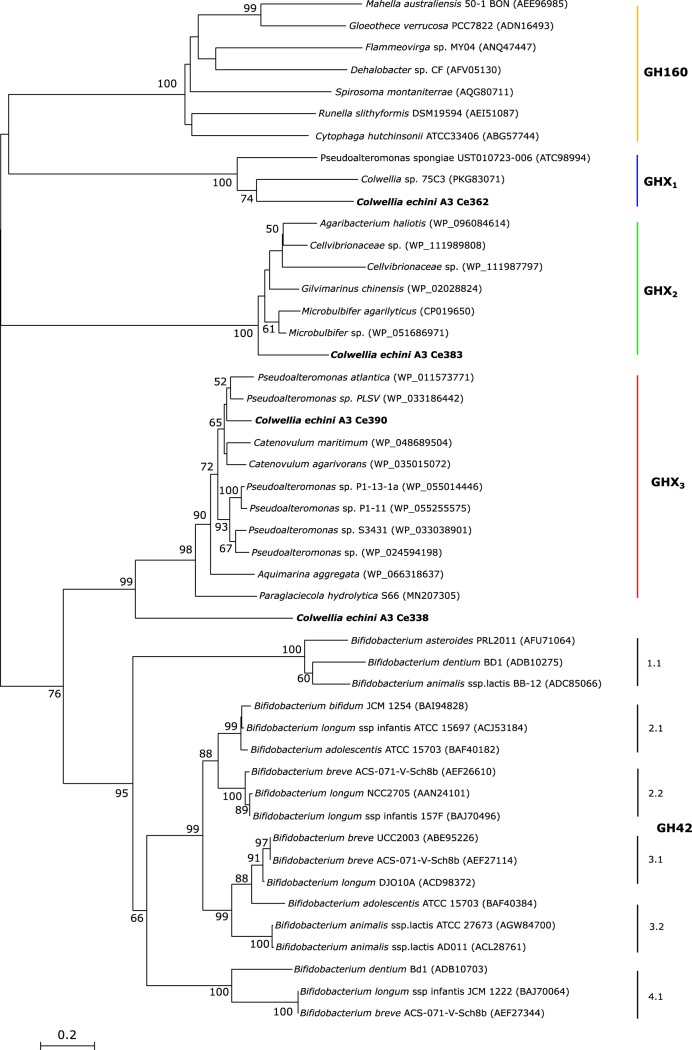
Neighbor-joining tree showing phylogenetical relationships between Ce338, Ce362, Ce383, and Ce390 from C. echini A3^T^ and characterized GH42 and GH160 β-galactosidases. Characterized sequences of Ph1657 furcellaranases from P. hydrolytica S66 that hydrolyze neocarratetraose monosulfate and related sequences were retrieved from the NCBI database. Numbers at nodes represent bootstrap values and are shown as percentages of 1,000 replicates; only values of >50% are shown. Vertical bars to the right indicate the GH160 clade and the six subgroups of GH42, 1.1 to 4.1, according to Viborg et al. (50). Data representing GHX_1_ to GHX_3_ indicate glycoside hydrolase sequences that may represent novel GH families. Ce338, Ce362, Ce383, and Ce390 from this work were found in separate clades together with other uncharacterized proteins retrieved from the NCBI database. Bar, 0.2 changes per position.

**FIG 8:**
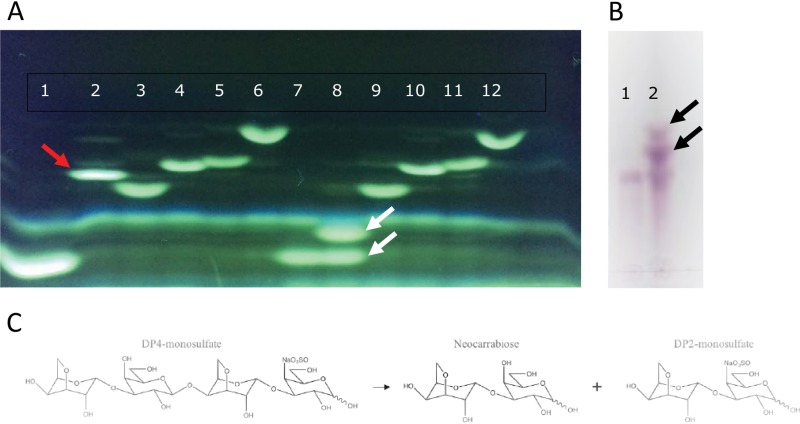
**(**A) FACE analysis showing 0.1% (wt/vol) neocarraoligosaccharides before and after digestion with Ce390. Lanes 1 and 7, DP2 monosulfate (monoS); lanes 2 and 8, DP4 monoS; lanes 3 and 9, DP4 diS; lanes 4 and 10; DP6 triS; lanes 5 and 11, DP6 tetraS; lanes 6 and 12, DP8 tetraS. Lanes 1 to 6 represent results obtained without enzyme; lanes 7 to 12 represent results obtained with Ce390 enzyme. Arrows indicate the position of DP4 monosulfate before (red) and after (white) digestion with Ce390. (B) TLC analysis of DP4 monosulfate before (lane 1) and after (lane 2) digestion with Ce390. Black arrows point to hydrolysis products. (C) Interpretation of activity of Ce390 on DP4 monosulfate.

In addition to GH16 and GH82 enzymes, bioinformatic analyses identified four additional open reading frames (ORFs; *Ce338*, *Ce362*, *Ce383*, and *Ce390*) that encode putative glycoside hydrolases with low similarity to known CAZymes. Phylogenetic analyses showed that Ce338 and Ce390 protein sequences were distantly related (less than 17% identity) to those of characterized lactose-specific β-galactosidases from GH42 and of newly characterized GH160 enzymes (Fig. 7). However, a BLASTp search revealed that sequences similar to those of Ce338 and Ce390 were discovered in several marine bacteria, including P. hydrolytica S66^T^ (10). Biochemical analysis showed that recombinant Ce390 hydrolyzed neocarraoligosaccharides such as neocarratetraose 41-*O*-monosulfate (DA-G-DA-G4S), neocarratetraose 41,43-*O*-disulfate (DA-G4S-DA-G4S), and neocarrahexaose 24,41,43,45-*O*-tetrasulfate (DA-G4S-DA2S-G4S-DA-G4S) to the corresponding unsulfated and sulfated neocarrabioses (Fig. 8; see also Fig. S2); lactose was not hydrolyzed (data not shown). As argued by Schultz-Johansen et al. (10), this GH42/GH160-like enzyme could be the enzyme that was described by McLean and Williamson (27), who published a similar enzyme activity from Pseudoalteromonas carrageenovora Psc^T^ and named the enzyme “neocarratetraose 4-*O*-monosulphate β-hydrolase.” Although BLASTP indicated a distant relationship with GH42 β-galactosidases (Table S2), the level of identity between GH42 (and GH160) enzymes and *Colwellia* enzymes Ce338 and Ce390 was low. Homologs to Ce338 and Ce390 and to Ce362 and Ce383 were discovered in other marine bacteria (Fig. 7), but since no enzyme activity could be ascribed to Ce362 or Ce383 and since no structures were known for any of the enzymes, more-detailed phylogenetic analyses were not possible.

10.1128/mSphere.00792-19.2 FIG S2 TLC analysis of neocarraoligosaccharides digested with Ce390. (A) Neocarratetraose 41-*O*-monosulfate (DA-G-DA-G4S) digestion. Lane 1, no enzyme; lane 2, E. coli extract with Ce390; lane 3, E. coli extract with empty vector. (B) Neocarratetraose 41,43-*O*-disulfate (DA-G4S-DA-G4S) digenstion. Lane 4, no enzyme; lane 5, E. coli extract with Ce390; lane 6, E. coli extract with empty vector. (C) neocarrahexaose 24,41,43,45-*O*-tetrasulfate (DA-G4S-DA2S-G4S-DA-G4S) digestion. lane 7, no enzyme; lane 8, E. coli extract with Ce390; lane 9, E. coli extract with empty vector. (D) Interpretation of results. Download FIG S2, PDF file, 0.1 MB. Copyright © 2020 Christiansen et al. 2020 Christiansen et al. This content is distributed under the terms of the Creative Commons Attribution 4.0 International license.

### Model for carrageenan utilization by C. echini A3^T^.

**FIG 9:**
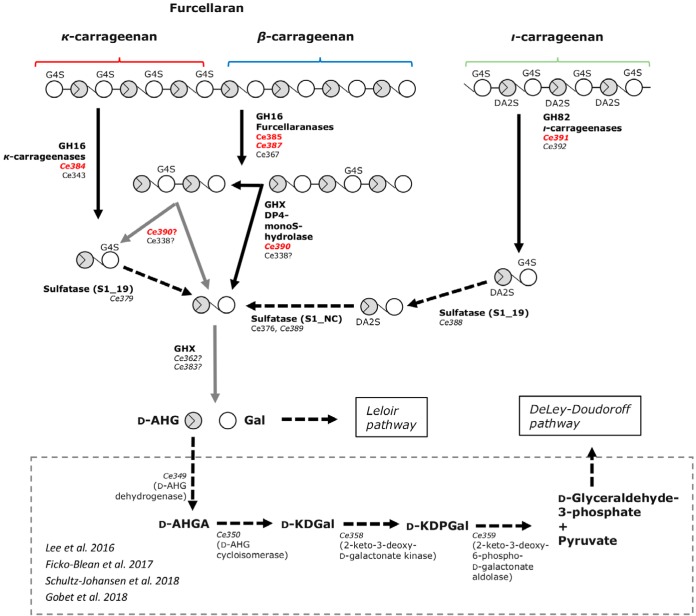
Pathway for degradation of β/κ-carrageenan (furcellaran), κ-carrageenan, and *ι-*carrageenan by C. echini A3^T^. Functionally characterized enzymes are shown in red, whereas italics indicate enzymes that were upregulated in native C. echini A3^T^ cultivated with κ-carrageenan. Black arrows indicate reactions that were documented through functional and/or bioinformatics analyses. Gray arrows indicate hypothesized enzyme activities that are yet to be confirmed. Data from Lee et al. (30), Ficko-Blean et al. (20), Schultz-Johansen et al. (10), and Gobet et al. (29) are indicated.

In addition to genes encoding furcellaranases, *ι-* and κ-carrageenases, and GH42/GH160-like enzymes, the CAR PUL gene cluster contained six genes encoding putative sulfatases (28) and enzymes expected to be involved in the final catabolism of hydrolysis products of carrageenan breakdown. Transcriptomics analysis showed that several of these sulfatases and auxiliary enzymes were upregulated under conditions of C. echini A3^T^ cultivation in the presence of κ-carrageenan (Fig. 1; see also Table S3). By combining functional studies, phylogenetical analyses, and transcriptomics, we were able to propose a model for how C. echini A3^T^ degrades hybrid β/κ-carrageenan, κ-carrageenan, and *ι-*carrageenan (Fig. 9). Furcellaranases Ce385 and Ce387 and possibly Ce367 degrade furcellaran to neocarratetraose-mono-sulfate (DA-G4S-DA-G/DA-G-DA-G4S) and neocarrahexaose-mono-sulfate (DA-G4S-DA-G-DA-G/DA-G-DA-G4S-DA-G/DA-G-DA-G-DA-G4S). Ce390 (and probably Ce338), upregulated in transcriptomics, subsequently degrades neocarratetraose-mono-sulfate to neocarrabiose (DA-G) and neocarrabiose-mono-sulfate (DA-G4S). Sulfatase Ce379 is upregulated during cultivation of strain A3^T^ on carrageenan and shows 62% identity to the S1_19 sulfatase PCAR9_p0034 (Table S4) that removes sulfate from galactose-4-sulfate in κ-type carrageenans in P. carrageenovora 9^T^ (29). Thus, we propose that Ce379 has a similar function in strain A3^T^ and converts DA-G4S to unsulfated carrabiose, namely, DA-G.

10.1128/mSphere.00792-19.7 TABLE S3 Transcriptomics analysis of C. echini A3^T^ cultivated with κ-carrageenan and glucose as carbon sources. Genes located in CAR PUL are shown in the white rows and genes in AGA PUL in the gray rows. logFC data represent the fold upregulation of genes measured when the bacterium was grown in carrageenan (Car-1 to Car-3) relative to expression in glucose (Glu-1 to Glu-3). logCPM data represent log counts per million; PValue, probability value; FDR, false-discovery rate. Download Table S3, DOCX file, 0.04 MB. Copyright © 2020 Christiansen et al. 2020 Christiansen et al. This content is distributed under the terms of the Creative Commons Attribution 4.0 International license.

10.1128/mSphere.00792-19.8 TABLE S4 Characteristics of sulfatases encoded by C. echini A3^T^. The sulfatase type was determined using the prediction program SulfAtlas (28); prediction of lipopeptides was carried out with Pred-Lipo for Gram-positive bacteria (44) and LipoP for Gram-negative bacteria (47); signal sequences were predicted with Signal P 5.0 (45, 46). Comparative analyses of sulfatases from C. echini A3^T^ and P. carrageenovora 9^T^ were carried out with BLASTp and Clustal W. Download Table S4, DOCX file, 0.01 MB. Copyright © 2020 Christiansen et al. 2020 Christiansen et al. This content is distributed under the terms of the Creative Commons Attribution 4.0 International license.

The κ-carrageenase Ce384 and Ce372 were upregulated in transcriptomics (log fold change [logFC] values of 3.69 and 3.03, respectively), and Ce384 was shown to degrade κ-carrageenan to DA-G4S, which would subsequently be desulfated to DA-G by the G4S sulfatase Ce379 as described above.

A pathway for the degradation of *ι-*carrageenan in strain A3^T^ showed similarity to the pathways in P. carrageenovora 9^T^ (29) and Z. galactanivorans (20). The *ι-*carrageenase Ce391 and possibly Ce392 hydrolyze *ι-*carrageenan to *ι-*neocarrabiose (DA2S-G4S), which subsequently may be desulfated by a DA2S sulfatase and a G4S sulfatase. A search for these sulfatases in the genome sequence of A3^T^ showed that the S1_19 sulfatase Ce388 displayed 56% and 50% identities to the G4S sulfatases from P. carrageenovora 9^T^ (PCAR_p0023, S1_19) and Z. galactanivorans (ZGAL_3145, S1_19), respectively (Table S4), which previously have been reported to cleave the 4-sulfate in *ι-*carrageenan. Subsequent desulfation of the 2-sulfate may be carried out by Ce376 (S1_NC) and Ce389 (S1_NC), which display 58% and 61% identity to a *ι-*specific DA2S sulfatase from P. carrageenovora 9^T^ (PCAR_p0022; S1_NC) (29). Both *ι-*carrageenases (Ce391 and Ce392) and the two sulfatases Ce388 (S1_19) and Ce389 (S1_NC) were upregulated in transcriptomics analysis. The CAR PUL encoded two more putative sulfatases, Ce363 (S1_7) and Ce364 (S1_19), with less than 31% and 35% identity to known sulfatases (Table S4).

Thus, the net result of degradation of furcellaran and κ- and *ι-*carrageenan is hypothesized to be neocarrabiose (DA-G). In order for neocarrabiose to enter the central metabolism, this disaccharide must be hydrolyzed further to galactose and d-AHG (3,6-anhydro-d-galactose). Ficko-Blean et al. (20) showed that Z. galactanivorans encodes GH127 and GH129 enzymes capable of hydrolysing d-AHG from neocarraoligosaccharides, and Schultz-Johansen et al. (10) reported that a GH127 enzyme similar to those of Z. galactanivorans was found in P. hydrolytica S66^T^. A search in the C. echini A3^T^ genome sequence for GH127 and GH129 enzymes produced negative results. However, as C. echini A3^T^ was able to grow with κ-carrageenan as the sole carbon source, the bacterium must contain a gene encoding an enzyme that hydrolyzes neocarrabiose to d-AHG and d-galactose.

Whereas d-galactose enters directly into the Leloir pathway, d-AHG must be processed further before entering into the central metabolism. Genes similar to those encoding the four d-AHG-converting enzymes that have been associated with catabolism of d-AHG (20, 29, 30) were identified in strain A3^T^; thus, it is proposed that strain A3^T^ is able to utilize both d-AHG and d-galactose. In Cellulophaga lytica LIM-21, Pseudoalteromonas atlantica T6c, *Epulopiscium* sp., Z. galactanivorans, and P. hydrolytica S66^T^, the four d-AHG-converting enzymes were organized in an operon-like structure (Fig. S4A). However, in C. echini A3^T^ and another agarolytic *Colwellia* species, C. agarivorans QM50^T^, the first two enzymes, 3,6-anhydro-d-galactose dehydrogenase and 3,6-anhydro-d-galactonate cycloisomerase, were encoded by two overlapping reading frames (in strain A3^T^ *Ce349* and *Ce350*), indicating that the genes were cotranscribed. The last two enzymes, 2-keto-3-deoxy-d-galactonate kinase and 2-keto-3-deoxy-6-phospho-d-galactonate aldolase (in A3^T^ *Ce359* and *Ce358*), were similarly encoded by two ORFs separated by only four nucleotides, indicating that the two genes could also be cotranscribed. Transcriptomics analysis showed that all four genes were upregulated when strain A3^T^ was grown on carrageenan (Table S3). Thus, the net result of the reaction of enzymes Ce349, Ce350, Ce358, and Ce359 would be the conversion of d-AHG to pyruvate and d-glyceraldehyde-3-phosphate, which enter the central metabolism through the DeLey-Doudoroff pathway (Fig. 9).

10.1128/mSphere.00792-19.3 FIG S3 Analysis of reaction products by MALDI-TOF mass spectroscopy. Mass spectra were obtained with (a) κ-carrageenan plus Ce384, (b) furcellaran plus Ce384, (c) furcellaran plus Ce385, and (d) furcellaran plus Ce387. Expected molecular weights and identities of the peaks are indicated in the spectra. Download FIG S3, PDF file, 0.1 MB. Copyright © 2020 Christiansen et al. 2020 Christiansen et al. This content is distributed under the terms of the Creative Commons Attribution 4.0 International license.

10.1128/mSphere.00792-19.4 FIG S4 Organization of genes involved in catabolism of d-AHG (A) and l-AHG (B) in C. echini A3^T^ and related species. The color code for the enzymes is indicated below the gene map. The orders of enzyme reactions in the breakdown of d-AHG and l-AHG are as follows: (A) Ce349 (3,6-anhydro-d-galactose dehydrogenase) → Ce350 (3,6-anhydro-d-galactonate cycloisomerase) → Ce358 (2-keto-3-deoxy-d-galactonate kinase) → Ce359 (2-keto-3-deoxy-6-phospho-d-galactonate aldolase) and (B) Ce2850 (3,6-anhydro-l-galactose dehydrogenase) → Ce2847 (3,6-anhydro-l-galactonate cycloisomerase) → Ce2848 (2-keto-3-deoxy-l-galactonate dehydrogenase) → Ce2849 (2,5-diketo-3-deoxy-l-galactonate 5-reductase). Download FIG S4, PDF file, 0.1 MB. Copyright © 2020 Christiansen et al. 2020 Christiansen et al. This content is distributed under the terms of the Creative Commons Attribution 4.0 International license.

## DISCUSSION

The marine bacterium C. echini A3^T^ isolated from the gut of sea urchin utilizes agar and κ-carrageenan for growth (9). In this study, we performed bioinformatic, structural, and biochemical analyses of the enzymatic carrageenan degradation system in C. echini A3^T^. Among all of the sequenced genomes of *Colwellia* species, that of C. echini A3^T^ has the greatest number of CAZymes and other enzymes involved in degradation of agars and carrageenans. Likewise, the genome of C. agarivorans QM50^T^ encodes many agarolytic enzymes, which correlates with its ability to hydrolyze agar (13). Thus, the CAZyme repertoires of these two *Colwellia* species reflect their physiological function as red alga degraders.

Algal polysaccharide-degrading bacteria are common in the marine environment: several reports previously described CAZyme-encoding *Bacteroidetes* (20, 31–34) and gammaproteobacteria (10, 29, 35). However, the genetic organizations of CAZymes and other enzymes involved in algal polysaccharide degradation may differ considerably. In P. hydrolytica S66^T^, all genes involved in utilization of agar, furcellaran, and κ-carrageenan are localized in one large ∼167-kb PUL (10). In P. carrageenovora 9^T^ and *Alteromonas* sp. 76-1, algal hydrolyzing enzymes are encoded on large plasmids (29, 35), and sometimes CAZymes are scattered on the chromosomes; e.g., agarases are scattered across four contigs in C. agarivorans (data not shown). Here, we show that genes necessary for degradation of different types of carrageenans are located in one ∼86,000-bp region, CAR PUL, and that agarolytic genes are found on another ∼92,000-bp region (AGA PUL). As discussed by Gobet et al. (29) and Schultz-Johansen et al. (10), the PUL structure of gammaproteobacteria shows a resemblance to the PULs described from *Bacteroidetes*. Putative transporters and TonB-dependent receptors, proposed to be analogous to the canonical SusC/SusD sensor/regulator system in *Bacteroidetes* PUL structures (36, 37), were found in both C. echini PULs, and transcriptomics showed that genes in CAR PUL were jointly regulated when C. echini was cultivated with κ-carrageenan as the sole carbon source. Gene activity in the AGA PUL was similarly shown to be jointly regulated (data are to be presented elsewhere).

The degradation of κ- and *ι-*carrageenan to neocarrabiose (DA-G) is catalyzed through the combined activities of κ- and *ι*-carrageenases (GH16_17, GH82, GH42-like, etc.) and of κ- and *ι*-specific sulfatases (S1_19) as shown for P. hydrolytica S66^T^ (10) and Z. galactanivorans Dsij^T^ (20) and as hypothesized for P. carrageenovora 9^T^ (29). Degradation of the β/κ-carrageenan furcellaran is initiated by specific GH16 endolytic enzymes that recognize only partially sulfated carrageenan. The products released from hydrolysis of furcellaran were previously proposed to be DP4-monosulfate and DP6-monosulfate (10). Here, we show that enzymes Ce385 and Ce387 from strain A3^T^ are such GH16_13 furcellaranases. We here show by mass spectrometry (MS) that the products are neocarratetraose-mono-sulfate and neocarrahexaose-mono-sulfate.

Neocarrabiose is a common key metabolite seen in the degradation of carrageenans in all bacteria investigated so far. However, bacteria metabolize neocarrabiose differently among different species. In Z. galactanivorans Dsij^T^, GH127 and GH129 enzymes hydrolyze neocarrabiose to d-AHG and galactose, which then enter the central metabolism (20). Mining the genome of P. hydrolytica S66^T^ revealed a similar (60% to 62% identity) GH127-encoding gene (10), but previous searches for GH127 and GH129 enzymes in P. carrageenovora 9^T^ and C. echini A3^T^ gave negative results. This led Gobet et al. (29) to conclude that P. carrageenovora is unable to release 3,6-anhydro-d-galactose from carrageenan degradation products. We observed that C. echini A3^T^ was able to grow with κ-carrageenan as the sole carbon source, and the fact that all genes necessary for degradation of κ-carrageenan to neocarrabiose and for the subsequent catabolism of d-AHG are found in CAR PUL led us to look for novel neocarrabiose-hydrolyzing enzymes in this gene cluster. Candidates for this activity could be the putative hydrolases Ce362 and Ce383 (separately or together). Both enzymes were shown to have carbohydrate binding domains by HHpred analysis, and they displayed low similarity to known GH enzymes, indicating that they may represent new GH families. BLAST and phylogenetical analyses indicated that both enzymes might be distantly related to known β-galactosidases affiliated with GH42 and GH160 families and to the GHX neocarratetraose monosulfate hydrolase described here, indicating that Ce362 and/or Ce383 might hydrolyze galactose-containing sugars such as neocarrabiose. Results of Pred-Lipo, Signal P, and Lipo P analyses indicated that Ce383 could be cytoplasmic whereas Ce362 might be anchored in the membrane. The predicted intracellular localization supports the hypothesis that Ce362 and/or Ce383 might be the missing 3,6-anhydro-d-galactosidase, as neocarrabiose is believed to be transported into the periplasm or cytoplasm in analogy with neoagarobiose (38, 39). However, functional analysis was not possible because expression of both genes in E. coli failed to give soluble, active enzymes. Once neocarrabiose has been transported into the cell and is hydrolyzed to d-AHG and galactose, galactose enters the general metabolism via the Leloir pathway, and d-AHG is converted to pyruvate and to d-glyceraldehyde-3-phosphate, which enters the metabolism via the DeLey-Doudoroff pathway as reported previously (10, 20, 29, 30).

## MATERIALS AND METHODS

### Bacterial strains and growth conditions.

C. echini A3^T^ was isolated and cultivated as described by Christiansen et al. (9). Escherichia coli TOP10 (Novagen) was used for cloning, and E. coli BL21(DE3) (Novagen) and E. coli BL21(DE3) Δ*lac*Z, kindly provided by Jin-Ho Seo from Seoul National University, South Korea, was used for expression studies.

### DNA isolation, gene cloning, and expression of recombinant enzymes.

Extraction of genomic DNA, PCR with gene-specific primers (see Table S5 in the supplemental material), and cloning of genes into plasmids pET9a-USER-1 and pET9a-USER-2 were carried out as described previously (10). Expression of recombinant enzymes was carried out in ZYP-5052 autoinduction medium (40) supplemented with appropriate antibiotics, and cells were disrupted in a FastPrep-24 5G bead beater (MP Biomedicals) as described by Schultz-Johansen et al. (10). Recombinant, His-tagged enzymes were subsequently applied to a HisTrap FF column (GE Healthcare, Uppsala, Sweden) charged with 100 mM NiSO_4_. After a washing step performed with 50 mM imidazole, the bound proteins were eluted with a linear gradient of imidazole ranging from 50 to 700 mM. Final protein concentration and purity were determined with a NanoDrop spectrophotometer (Thermo Scientific, Illkirch, France) and by SDS/PAGE, respectively. Extracts from E. coli cells treated with an empty vector were processed and analyzed in parallel as negative controls.

10.1128/mSphere.00792-19.9 TABLE S5 Primer sequences for amplification of genes in the carrageenolytic gene cluster. Two expression vectors were used, pET9a.USER-1 and pET9a.USER-2 (10). Download Table S5, DOCX file, 0.01 MB. Copyright © 2020 Christiansen et al. 2020 Christiansen et al. This content is distributed under the terms of the Creative Commons Attribution 4.0 International license.

### Enzyme reactions and activity visualization.

Crude cell lysates were analyzed in a 3-methyl-2-benzothiazolinone hydrazine (MBTH) reducing end sugar assay (41). *κ*-Carrageenan, *ι*-carrageenan, *λ*-carrageenan, agar (Sigma-Aldrich), porphyran (prepared as described previously [23]), furcelleran (Est-Agar, Estonia), and agarose (Invitrogen) were used as the substrates (0.1% [wt/vol]) in a 50-μl reaction mixture with 1 μl crude cell lysate.

Thin-layer chromatography (TLC) analysis of enzyme activity was carried out in total volumes of 50 μl containing 10 μl crude cell lysate and 0.1% (wt/vol) polysaccharide. Assays performed with neocarraoligosaccharides (Dextra, United Kingdom) were carried out volumes of 1 μl crude enzyme and 2 μg/μl (wt/vol) of the respective oligosaccharide substrates in a total reaction volume of 12 μl. All reaction mixtures were incubated overnight. The enzyme reaction mixtures (6 μl) were spotted onto a silica gel 60 TLC plate (Merck). Plates were run twice in *n*-butanol-acetic acid-water (2:1:1 [vol/vol/vol]). Visualization of the plates was performed with 5-methylresorcinol monohydrate–5% (wt/vol) H_2_SO_4_, and development on a heating plate was performed for approximately 5 min.

For enzymatic reactions analyzed by fluorophore-assisted carbohydrate electrophoresis (FACE) (42), 5-μl volumes of crude cell lysates were used with a final concentration of 0.1% (wt/vol) polysaccharide. Enzyme reactions were performed with oligosaccharides as described above. Reaction mixtures were incubated overnight, and reactions were terminated at 90°C for 10 min followed by centrifugation (17,000 × g, 10 min). The supernatants were dried in a speed-vacuum centrifuge. Samples were labeled with 2 μl 8-aminonaphthalene-1,3,6-trisulfonate (ANTS) solution (0.15 M ANTS dissolved in acetic acid-water [3:17 {vol/vol}]) and with 5 μl freshly prepared 1 M sodium cyanoborohydride–dimethyl sulfoxide (DMSO) (38). After incubation overnight at 37°C, 25 μl glycerol (25%) was added and 5 to 10 μl was loaded onto a 6% stacking and 27% running polyacrylamide gel. The gel was subjected to electrophoresis at 4°C at 200V for 2 h. Hydrolysis was visualized under UV light.

### DNA sequence analyses and *in silico* analysis.

Automatic annotation was performed online in the Rapid Annotation using Subsystem Technology (RAST) server (43). Correlations between gene and protein names reported in this work and locus tags in GenBank are shown in Table S6. A more thorough search and identification of CAZymes and sulfatases were carried out using hidden Markov model searches as described previously by Schultz-Johansen et al. (10). Selected gene sequences from RAST were imported into CLC Main Workbench 7.0 (Qiagen) for primer design. Similarity searches and predictions of conserved domains were performed by the use of BLASTp searches against the NCBI (https://www.ncbi.nlm.nih.gov) Protein Data Bank database (PDB), Swiss-Prot, and the nonredundant protein database (nr) and the Conserved Domain Database (CDD). Prediction of signal peptides and lipoproteins was performed with Pred-Lipo (44), SignalP 4.1 (45, 46), and LipoP 1.0 (47). All alignments were made in CLC Main Workbench 7.0, and phylogenetic trees were constructed in MEGA7 (48). Sequences were retrieved from the nonredundant database using C. echini A3^T^ genes or sequences from structurally determined proteins. *In silico* folding was performed using SWISS-MODEL (https://swissmodel.expasy.org) (51), and model quality was assessed using the QMEAN server (http://swissmodel.expasy.org/qmean) (52); models with QMEANDisCo values of <0.6 were discarded (51). Additionally, local quality estimate was used and only residues with a local quality score of >0.75 are discussed. Structural models were superimposed using the “super” function in PyMOL (The PyMOL Molecular Graphics System, Version 2.0; Schrödinger, LLC).

10.1128/mSphere.00792-19.10 TABLE S6 Correlation between names and function of proteins in this work and the corresponding genes in scaffold 5 of the Colwellia echini A3^T^ genome sequence, PJAI00000000.2. Download Table S6, DOCX file, 0.01 MB. Copyright © 2020 Christiansen et al. 2020 Christiansen et al. This content is distributed under the terms of the Creative Commons Attribution 4.0 International license.

### RNA-seq analysis.

C. echini A3^T^ was grown in marine minimal broth (MMB) containing 2.3% (wt/vol) aquarium sea salt mix (Instant Ocean Sea Salts; Aquarium Systems, Mentor, OH, USA), 0.1% (wt/vol) yeast extract, 0.05% (wt/vol) NH_4_Cl, and 10 mM Tris-HCl buffer (pH 7.4). d-Glucose (DaeJung, South Korea) or κ-carrageenan (Tokyo Chemical Industries, Japan) (0.2% [wt/vol]) was supplemented as the sole carbon source. All experiments were carried out in triplicate. Cells for transcriptome sequencing (RNA-seq) analysis were cultivated in MMB at 20°C and 180 rpm. Overnight cultures were diluted in MMB containing either 0.2% (wt/vol) d-glucose or 0.2% (wt/vol) κ-carrageenan, and when the optical density at 600 nm (OD_600_) reached 0.8, cells were harvested by centrifugation at 4°C. Cell pellets were resuspended in 1 ml of TRIzol reagent (Invitrogen) and incubated 2 min at room temperature for cell lysis. After complete lysis, cell lysates were mixed with 200 μl of chloroform and subjected to brief vortex mixing. Phase separation was facilitated by centrifugation at 12,500 × *g* for 5 min, and the aqueous phase was separated. A half-volume of isopropanol (Sigma-Aldrich, St. Louis, MO, USA) was added to the separated aqueous phase and loaded onto an RNeasy minicolumn (Qiagen, Inc.). The column was washed with 70% ethanol, and RNA was eluted with RNase-free water. A Qubit RNA BR assay kit (Invitrogen) was used to determine the RNA quantity. The quality of the RNA was determined using a Qsep1 Bio-fragment analyzer (BiOptic Inc.) equipped with an RNA cartridge. Extracted RNA was stored at –80°C until further use. Before the library preparation, mRNA enrichment was performed using a Ribo-Zero rRNA removal kit for bacteria (Illumina, Inc.). Enriched mRNA samples were purified using Agencourt AMPure XP beads (Beckman Coulter, Inc.). The RNA-seq library was prepared using a NEBNext Ultra RNA library preparation kit for Illumina (New England Biolabs, USA) according to the instructions of the manufacturer. Sequencing was performed using an Illumina MiSeq platform (Illumina, Inc.) and Illumina MiSeq reagent kit V3 (300 bp by 2 paired end).

Quality control and trimming of Illumina sequenced reads were performed with TrimGalore ver. 0.5.0 (https://www.bioinformatics.babraham.ac.uk/projects/trim_galore/). Illumina universal adapter sequences and the index sequences were trimmed. Reads shorter than 40 bp were discarded, and the bases with Q values of <20 were trimmed from the 3′ and 5′ ends of reads. Reads were mapped to the C. echini A3^T^ genome (GenBank accession no. PJAI00000000.2) by the use of Bowtie2 software. SAMtools was used to convert the alignment files into BAM files. The numbers of reads mapped to the predicted coding DNA sequence (CDS) were analyzed using the Bioconductor package (49). Differential gene expression analysis was performed with the edgeR package in Bioconductor. Genes with a *P* value of <0.05 and a |logFC| value of >2 were considered to be differentially expressed (Table S3).

### Qualitative analysis of reaction products by MALDI-TOF.

For qualitative analysis of reaction products, MALDI-TOF MS was carried out using 2,5-dihydroxybenzoic acid (DHB) as the matrix. DHB was dissolved in 50:50 (vol/vol) acetonitrile and water containing 0.5% trifluoroacetic acid (TFA) at a concentration of 10 mg/ml. The aliquot of the enzymatic reaction products (2 μl) was placed on the target plate and mixed with DHB. The mass spectra were obtained using a Bruker UltrafleXtreme MALDI MS instrument (Bremen, Germany) equipped with a SmartBeam II laser.

### Data availability.

The draft genome sequence of C. echini A3T is available from GenBank (accession number PJAI00000000.2).
