# Supplementary material for: A Multifunctional Polysaccharide Utilization Gene Cluster in Colwellia echini Encodes Enzymes for the Complete Degradation of κ-Carrageenan, ι-Carrageenan, and Hybrid β/κ-Carrageenan
Source: mSphere. 2020 Jan 8;5(1):e00792-19. doi: 10.1128/mSphere.00792-19 (PMC6952198; doi:10.1128/mSphere.00792-19)
Supplement: TABLE S1 [file mSphere.00792-19-st001.docx]

**Table S1**

| **A** |  |  |  |  |  |
| --- | --- | --- | --- | --- | --- |
| CAZy  family | *C. echini*  A3^T^ | *C. agarivorans*  QM50^T^ | *C. psychrerythraea*  34H^T^ | *C. piezophila*  Y223G^T^ | *C. chukchiensis*  BCw111^T^ |
| AA | 16 | 6 | 12 | 4 | 7 |
| CBM | 86 | 30 | 29 | 30 | 12 |
| CE | 34 | 30 | 28 | 52 | 29 |
| GH | 100 | 84 | 48 | 82 | 19 |
| GT | 37 | 27 | 28 | 34 | 19 |
| PL | 19 | 8 | 5 | 6 | 4 |
| Total CAZymes | 292 | 185 | 150 | 208 | 90 |
| Sulf | 11 | 35 | 16 | 49 | 4 |
|  |  |  |  |  |  |
| **B** |  |  |  |  |  |
| CAZy  family | *C. echini*  A3^T^ | *C. agarivorans*  QM50^T^ | *C. psychrerythraea*  34H^T^ | *C. piezophila*  Y223G^T^ | *C. chukchiensis*  BCw111^T^ |
| GH16 | 9 | 8 | 3 | 4 | 1 |
| GH50 | 4 | 4 | 0 | 2 | 0 |
| GH82 | 2 | 0 | 0 | 0 | 0 |
| GH86 | 1 | 3 | 0 | 0 | 0 |
| GH96 | 2 | 0 | 0 | 0 | 0 |
| GH117 | 1 | 6 | 0 | 0 | 0 |
| CBM6 | 9 | 0 | 0 | 3 | 0 |
