# Supplementary material for: A Multifunctional Polysaccharide Utilization Gene Cluster in Colwellia echini Encodes Enzymes for the Complete Degradation of κ-Carrageenan, ι-Carrageenan, and Hybrid β/κ-Carrageenan
Source: mSphere. 2020 Jan 8;5(1):e00792-19. doi: 10.1128/mSphere.00792-19 (PMC6952198; doi:10.1128/mSphere.00792-19)
Supplement: TABLE S2 [file mSphere.00792-19-st002.docx]

**Table S2**

| ***C. echini* A3** | **GH family** | **Database** | **Predicted enzyme activity** | **Closest relative** | **% identity** | **Accession no.** |
| --- | --- | --- | --- | --- | --- | --- |
| Ce343 | GH16 | PDB  Swiss-Prot  nt | *κ-*carrageenase  *κ-*carrageenase  hypothetical protein | *Pseudoalteromonas carrageenovora*  *Pseudoalteromonas carrageenovora*  *Colwellia* sp. PAMC 21821 | 42  41  70 | 5OCQ  P43478.1  ARD46027 |
| Ce362 | GHX | PDB  Swiss-Prot  nt | hypothetical protein  hyalin  hypothetical protein | *Moritella marina*  *Strongylocentrotus purpuratus*  *Colwellia* sp. 75C3 | 15  15  38 | 4HMC_A  O76536.1  PKG83071 |
| Ce367 | GH16 | PDB  Swiss-Prot  nt | β-porphyranase  β-porphyranase  hypothetical protein | *Zobellia galactanivorans*  *Zobellia galactanivorans*  *Microbulbifer* sp. HZ11 | 27  27  74 | 4ATE  D7GXG0.1  WP_043316997 |
| Ce372 | GH16 | PDB  Swiss-Prot  nt | Laminarinase  Laminarinase  GH16 protein | *Rhodothermus marinus*  *Rhodothermus marinus*  *Thalassotalea* sp. ZS-4 | 26  26  67 | 3ILN  P45798.1  WP_136737284.1 |
| Ce383 | GHX | PDB  Swiss-Prot  nt | Alginase  50S ribosomal prot L17  hypothetical protein | *Pseudoalteromonas* sp. SM0524  *Tropheryma whipplei* str. Twist  *Microbulbifer* sp. HZ11 | 8  7  66 | 4Q8K  Q83G10.1  WP_051686971 |
| Ce384 | GH16 | PDB  Swiss-Prot  nt | *κ-*carrageenase  *κ-*carrageenase  GH 16 enzyme | *Pseudoalteromonas carrageenovora*  *Pseudoalteromonas carrageenovora*  *Catenovulum* sp. CCB-QB4 | 41  41  51 | 5OCQ  P43478.1  AWB65000 |
| Ce385 | GH16 | PDB  Swiss-Prot  nt | β-porphyranase  β-porphyranase  furcellaranase | *Zobellia galactanivorans*  *Zobellia galactanivorans*  *Paraglaciecola hydrolytica* S66^T^ | 29  29  40 | 4ATE  D7GXG0.1  WP_082768819 |
| Ce387 | GH16 | PDB  Swiss-Prot  nt | β-porphyranase  β-porphyranase  hypothetical protein | *Zobellia galactanivorans*  *Zobellia galactanivorans*  *Colwellia agarivorans* | 26  26  80 | 3JUU  D7GXF9.1  WP_077338640 |
| Ce390 | GHX | PDB  Swiss-Prot  nt | GH42 βgalactosidase  -  GH42 enzyme | *Geobacillus stearothermophilus*  No significant similarity found  *Pseudoalteromonas* sp. PLSV | 17  -  81 | 4OIF  -  WP_033186442 |
| Ce391 | GH82 | PDB  Swiss-Prot  nt | *ι-*carrageenase  *ι-*carrageenase  hypothetical protein | *Alteromonas* sp. ATCC 43554  Zobellia galactanivorans  *Pseudoalteromonas* sp. PLSV | 14  19  69 | 3LMW  Q9F284.1  WP_033186446 |
| Ce392 | GH82 | PDB  Swiss-Prot  nt | *ι-*carrageenase  *ι-*carrageenase  hypothetical protein | *Alteromonas* sp. ATCC 43554  *Zobellia galactanivorans*  *Pseudoalteromonas atlantica* T6c | 16  18  79 | 3LMW  Q9F284.1  WP_011573765 |
