# Supplementary figures and images for: A Multifunctional Polysaccharide Utilization Gene Cluster in Colwellia echini Encodes Enzymes for the Complete Degradation of κ-Carrageenan, ι-Carrageenan, and Hybrid β/κ-Carrageenan
Source: mSphere. 2020 Jan 8;5(1):e00792-19. doi: 10.1128/mSphere.00792-19 (PMC6952198; doi:10.1128/mSphere.00792-19)

**Fig. S1**

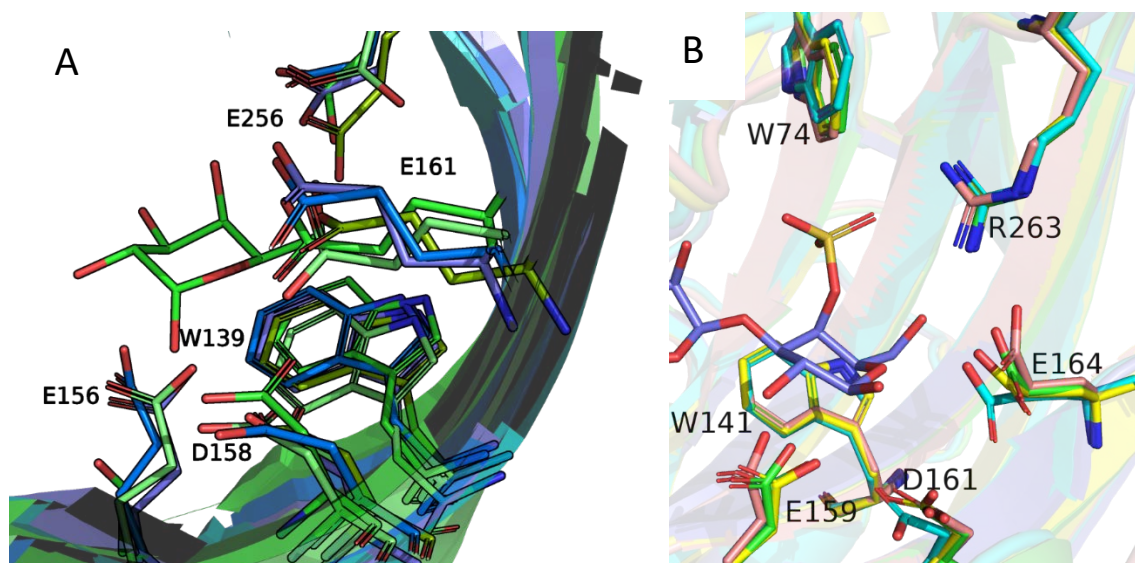

Supplement: FIG S1 [file mSphere.00792-19-sf001.pdf]

**Fig. S2**

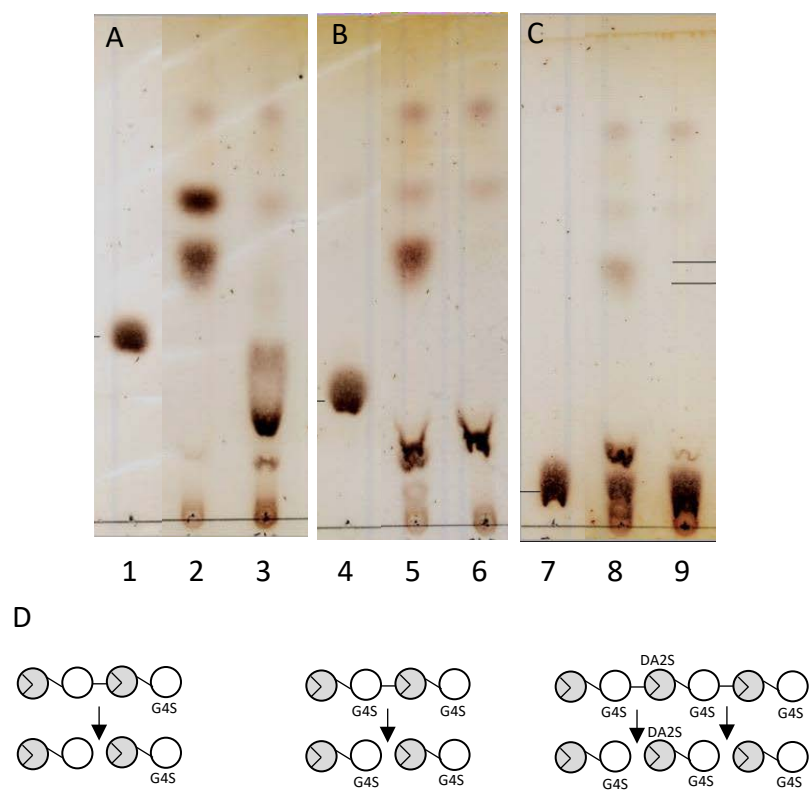

Supplement: FIG S2 [file mSphere.00792-19-sf002.pdf]

**Fig. S3**

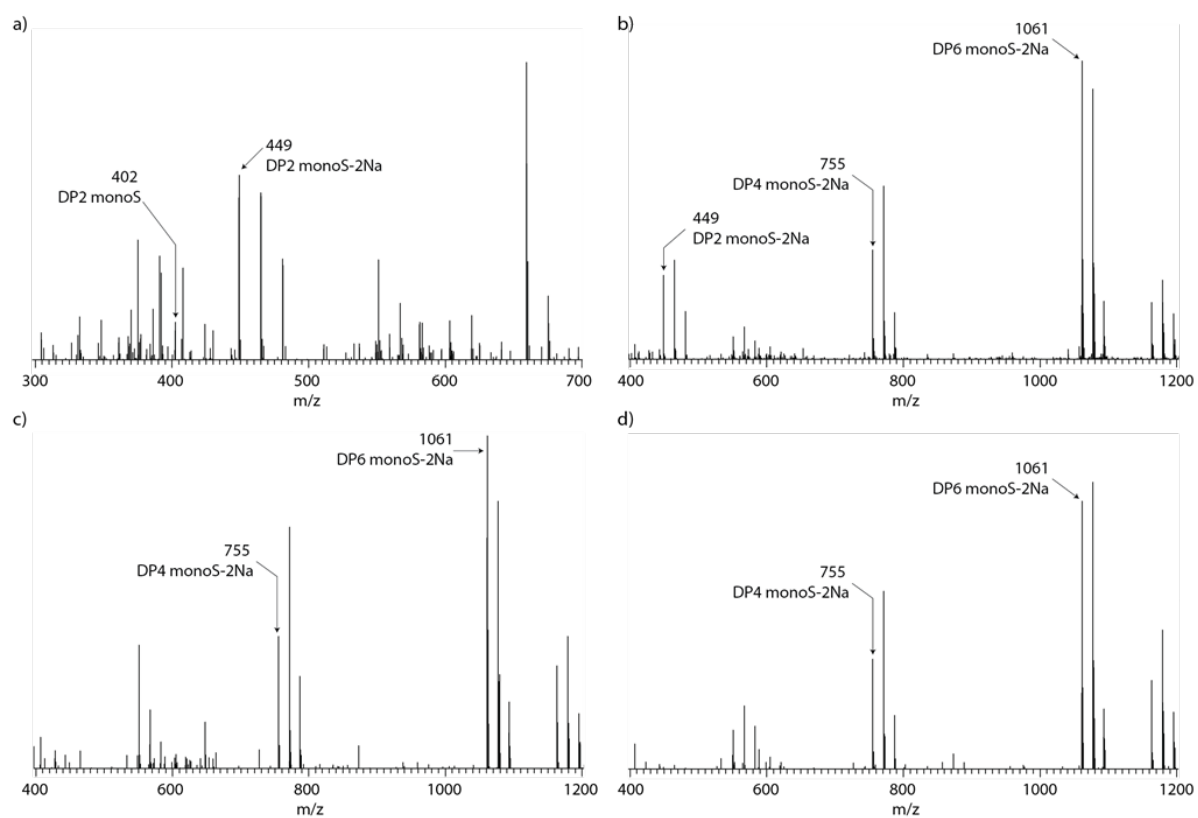

Supplement: FIG S3 [file mSphere.00792-19-sf003.pdf]

Fig. S4

A

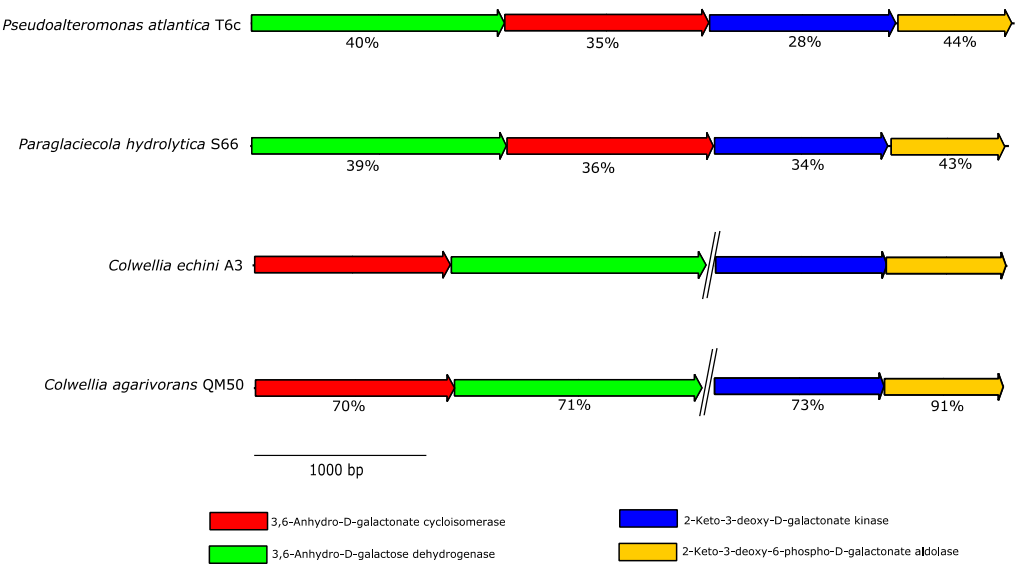

B

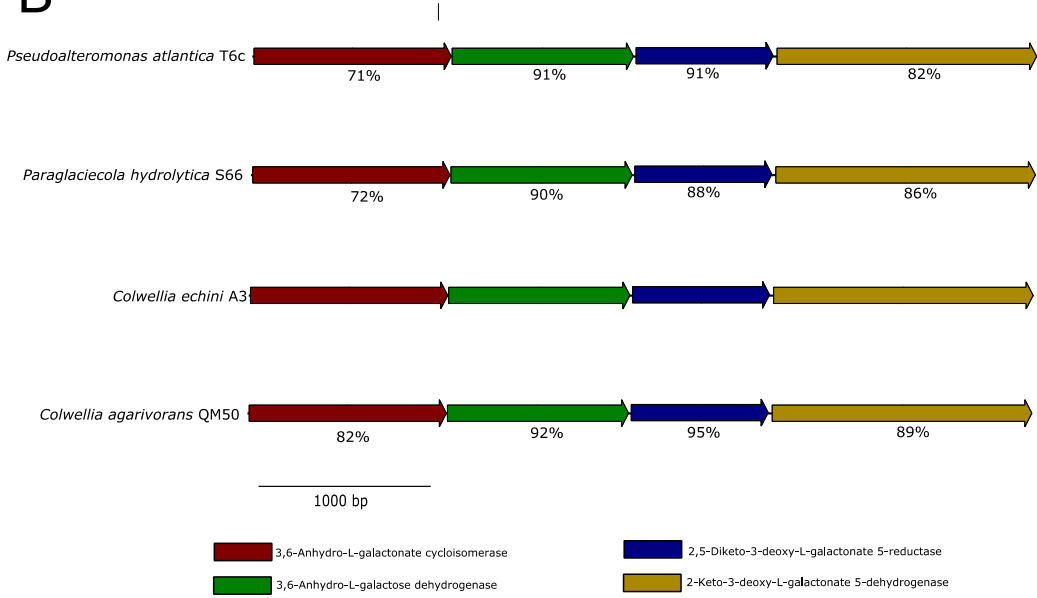

Supplement: FIG S4 [file mSphere.00792-19-sf004.pdf]
