# Supplementary material for: A Multifunctional Polysaccharide Utilization Gene Cluster in Colwellia echini Encodes Enzymes for the Complete Degradation of κ-Carrageenan, ι-Carrageenan, and Hybrid β/κ-Carrageenan
Source: mSphere. 2020 Jan 8;5(1):e00792-19. doi: 10.1128/mSphere.00792-19 (PMC6952198; doi:10.1128/mSphere.00792-19)
Supplement: TABLE S4 [file mSphere.00792-19-st004.docx]

**Table S4**

| *C. echini* A3^T^ | Sulfatase type^1^ | Signal P^2^ / Pred-Lipo^3^/Lipo P^4^ | *P. carrageenovora* | Sulfatase type^1^ | Identity (%) |
| --- | --- | --- | --- | --- | --- |
| Ce363 | S1_7 | + / - | PCAR9_P0022 | S1_NC | 19 |
| Ce364 | S1_19 | + / + | PCAR9_P0023 | S1_19 | 35 |
| Ce376 | S1_NC | + / + | PCAR9_P0022 | S1_NC | 58 |
| Ce379 | S1_19 | + / + | PCAR9_P0034 | S1_19 | 62 |
| Ce388 | S1_19 | + / + | PCAR9_P0023 | S1_19 | 56 |
| Ce389 | S1_NC | - / - | PCAR9_P0022 | S1_NC | 61 |
