# Supplementary material for: A Multifunctional Polysaccharide Utilization Gene Cluster in Colwellia echini Encodes Enzymes for the Complete Degradation of κ-Carrageenan, ι-Carrageenan, and Hybrid β/κ-Carrageenan
Source: mSphere. 2020 Jan 8;5(1):e00792-19. doi: 10.1128/mSphere.00792-19 (PMC6952198; doi:10.1128/mSphere.00792-19)
Supplement: TABLE S5 [file mSphere.00792-19-st005.docx]

**Table S5**

| **Gene** | **Primer name** | **Primer sequence** | **Vector** |
| --- | --- | --- | --- |
| Ce343 | Ce343_USER_F  Ce343_USER_R | 5’-GGCTTAAU GCTCAAAGTAAAACCATTAAGC-3’  5’-GGTTTAAU AGGCTTTTTCTTATAGACTCTGAC-3’ | pET9a.USER-1 |
| Ce367 | Ce367_67F  Ce367_2328R | 5’-ATATGGCU GTAGATATCCCACCTCCG-3’  5’-ACTTCCACU ACGAACATTCACCGTTAG-3’ | pET9a.USER-2 |
| Ce372 | Ce372_97F  Ce372_906R | 5’ACTTCCACU ACGAACATTCACCGTTAC-3’  5’-ACTTCCACU TTGCTTTTTATAAAAGCGTAC-3’ | pET9a.USER-2 |
| Ce383 | Ce383_58F  Ce383_3627R | 5’-ATATGGCU GTACCGGACAGACCTGTC-3’  5’-ACTTCCACU TTCATGTACGATACCCGAG-3’ | pET9a.USER-2 |
| Ce384 | Ce384_USER_F  Ce384_USER_R | 5’-GGCTTAAU GGCTCTAGTGACTCAGCT G-3’  5’-GGTTTAAU TGGCGCTCTTTTATAAACG-3’ | pET9a.USER-1 |
| Ce385 | Ce385_USER_F  Ce385_USER_R | 5’-GGCTTAAU ATGAAACTTAAAGCAAAAGC-3’  5’-GGTTTAAU TTTGGTTTTTCTTCGTG-3’ | pET9a.USER-1 |
| Ce387 | Ce387_USER_F  Ce387_USER_R | 5’-GGCTTAAU TCTGAAAATATATCTAGAAG-3’  5’-GGTTTAAU TTTTTCGAGTGCAATATTC-3’ | pET9a.USER-1 |
| Ce390 | Ce390_USER_F  Ce390_USER_R | 5’-GGCTTAAU GGAGTAGCTATTGCTTCAG-3’  5’-GGTTTAAU TTGCGGTTTCACTTCT-3’ | pET9a.USER-1 |
| Ce391 | Ce391_553F  Ce391_2262R | 5’-ATATGGCU ATAACGTTACCGCCTAGTG-3’  5’-ACTTCCACU ATCAGTTGCTTTCGTACC-3’ | pET9a.USER-2 |
| Ce392 | Ce392_868F  Ce392_2397R | 5’-ATATGGCU AATCCTGCTGATGGCA-3’  5’-ACTTCCACU ATTATCAAAACTTTCAGGTGTAAG-3’ | pET9a.USER-2 |
