# Supplementary material for: A Multifunctional Polysaccharide Utilization Gene Cluster in Colwellia echini Encodes Enzymes for the Complete Degradation of κ-Carrageenan, ι-Carrageenan, and Hybrid β/κ-Carrageenan
Source: mSphere. 2020 Jan 8;5(1):e00792-19. doi: 10.1128/mSphere.00792-19 (PMC6952198; doi:10.1128/mSphere.00792-19)
Supplement: TABLE S6 [file mSphere.00792-19-st006.docx]

**Table S6**

| **Gene** | **PJAI00000000.2**  **Scaffold 5 locus tag** | **Function** |
| --- | --- | --- |
| Ce338 | CWS31_006430 | GHX glycoside hydrolase |
| Ce343 | CWS31_006455 | GH16 *κ*-carrageenase |
| Ce345 | CWS31_006465 | TonB-dependent receptor |
| Ce349 | CWS31_006470 | d-AHG dehydrogenase |
| Ce350 | CWS31_006475 | d-AHG cycloisomerase |
| Ce358 | CWS31_006515 | 2-keto-3-deoxy-D-galactonate kinase |
| Ce359 | CWS31_006520 | 2-keto-3-deoxy-6-phospho-d-galactonate aldolase |
| Ce362 | CWS31_006535 | GHX glycoside hydrolase |
| Ce363 | CWS31_006540 | Sulfatase S1_7 |
| Ce364 | CWS31_006545 | Sulfatase S1_19 |
| Ce367 | CWS31_006560 | GH16 Furcellaranase |
| Ce372 | CWS31_006590 | GH16 *κ*-carrageenase |
| Ce376 | CWS31_006610 | Sulfatase S1_NC |
| Ce379 | CWS31_006625 | Sulfatase S1_19 |
| Ce383 | CWS31_006645 | GHX glycoside hydrolase |
| Ce384 | CWS31_006650 | GH16 *κ*-carrageenase |
| Ce385 | CWS31_006655 | GH16 Furcellaranase |
| Ce387 | CWS31_006665 | GH16 Furcellaranase |
| Ce388 | CWS31_006670 | Sulfatase S1_19 |
| Ce389 | CWS31_006675 | Sulfatase S1_NC |
| Ce390 | CWS31_006680 | GHX neocarratetraose monosulfate hydrolase |
| Ce391 | CWS31_006685 | GH82 *ι-*carrageenase |
| Ce392 | CWS31_006690 | GH82 *ι-*carrageenase |
